# MEG-PLAN: a clinical and technical protocol for obtaining magnetoencephalography data in minimally verbal or nonverbal children who have autism spectrum disorder

**DOI:** 10.1186/s11689-020-09350-1

**Published:** 2021-01-23

**Authors:** Emily S. Kuschner, Mina Kim, Luke Bloy, Marissa Dipiero, J. Christopher Edgar, Timothy P. L. Roberts

**Affiliations:** 1 Lurie Family Foundations MEG Imaging Center, Department of Radiology, The Children’s Hospital of Philadelphia, 2716 South Street, 5th Floor, Room 5251, Philadelphia, PA 19146 USA; 2 Department of Psychiatry, Perelman School of Medicine, University of Pennsylvania, Philadelphia, PA USA

**Keywords:** Autism spectrum disorder, Minimally verbal, Nonverbal, Intellectual disability, Magnetoencephalography, Imaging methodology, Compliance, Applied behavior analysis

## Abstract

**Background:**

Neuroimaging research on individuals who have autism spectrum disorder (ASD) has historically been limited primarily to those with age-appropriate cognitive and language performance. Children with limited abilities are frequently excluded from such neuroscience research given anticipated barriers like tolerating the loud sounds associated with magnetic resonance imaging and remaining still during data collection. To better understand brain function across the full range of ASD there is a need to (1) include individuals with limited cognitive and language performance in neuroimaging research (non-sedated, awake) and (2) improve data quality across the performance range. The purpose of this study was to develop, implement, and test the feasibility of a clinical/behavioral and technical protocol for obtaining magnetoencephalography (MEG) data. Participants were 38 children with ASD (8–12 years) meeting the study definition of minimally verbal/nonverbal language. MEG data were obtained during a passive pure-tone auditory task.

**Results:**

Based on stakeholder feedback, the *MEG Protocol for Low-language/cognitive Ability Neuroimaging* (MEG-PLAN) was developed, integrating clinical/behavioral and technical components to be implemented by an interdisciplinary team (clinicians, behavior specialists, scientists, and technologists). Using MEG-PLAN, a 74% success rate was achieved for acquiring MEG data, with a 71% success rate for evaluable and analyzable data. Exploratory analyses suggested nonverbal IQ and adaptive skills were related to reaching the point of acquirable data. No differences in group characteristics were observed between those with acquirable versus evaluable/analyzable data. Examination of data quality (evaluable trial count) was acceptable. Moreover, results were reproducible, with high intraclass correlation coefficients for pure-tone auditory latency.

**Conclusions:**

Children who have ASD who are minimally verbal/nonverbal, and often have co-occurring cognitive impairments, can be effectively and comfortably supported to complete an electrophysiological exam that yields valid and reproducible results. MEG-PLAN is a protocol that can be disseminated and implemented across research teams and adapted across technologies and neurodevelopmental disorders to collect electrophysiology and neuroimaging data in previously understudied groups of individuals.

## Background

Children on the autism spectrum^1^ are often involved in clinical research that uses non-invasive brain imaging technologies to study brain function and structure. Brain imaging research with paradigms requiring children to be awake has predominantly focused on youth who have age-appropriate cognitive and language abilities. It has been assumed that children with more limited cognitive and language performance will have difficulty completing neuroimaging protocols, given the need to remain still to reduce motion artifact, to tolerate novel sensory experiences, to inhibit stereotyped and repetitive behaviors and movements, and to understand/follow the unfamiliar directions associated with imaging [2].

Of note, however, is that 33% of individuals who have autism spectrum disorder (ASD) have cognitive abilities in the intellectual disability range [3], and individuals with ASD who are minimally verbal or nonverbal (MVNV) comprise ~ 40% of the ASD population [4, 5]. As such, restricting neuroimaging research to those without cognitive and language weaknesses excludes a large portion of individuals on spectrum. This restriction limits our understanding of brain function in ASD [6]. As an example, it is currently unknown if the structural and functional brain abnormalities observed in “higher functioning” ASD are also observed in “lower functioning” ASD and thus if brain imaging findings related to the etiology of ASD as well as treatment of ASD would remain valid across the wide range of people on the spectrum. It is important to note that MVNV language classification and intellectual disability represent different but often related weaknesses (these descriptors are not interchangeable but do often co-occur and thus both discussed below). Importantly, children with cognitive weaknesses and those who are minimally verbal or nonverbal are often excluded from neuroimaging research, a point that drives the development of the methods described in this paper.

Historically, structural MRI studies with children with ASD have relied on the use of anesthesia or other sedative strategies (e.g., [7–9]) to obtain scans without artifact and with some recent autism studies administering chloral hydrate to induce sleep in toddlers and young children [10, 11]. However, the side effects and associated risks of these drugs (e.g., adverse respiratory or cardiovascular events), as well as the potential that these drugs modify brain activity, are clear problems [12, 13]. Given the voluntary nature of research, as well as the desired use of brain imaging protocols that require an awake state and behavioral responses, researchers generally do not conduct ASD studies with sedation.

For studies where participants do not need to be awake, neuroimaging data may be obtained while the participant is asleep [14, 15]. This is a common strategy in infant imaging research [16, 17], and regularly used in studies examining infant siblings at high familial risk for ASD (e.g., [18, 19]). These studies, however, have their own challenges, such as infants waking in response to noise. And of course, sleep studies limit the opportunity to examine functional responses.

Given the limitations of neuroimaging with children on spectrum under sedation or while asleep, there is a need for novel behavioral and technical protocols to support the needs of individuals with ASD across the full spectrum undergoing brain imaging exams when awake, with an emphasis on strategies that provide brain imaging data from individuals with limited cognitive or language abilities.

Work in this area is growing. Historically, principles of applied behavior analysis (ABA) have been used in clinical settings to increase cooperation, and support habituation to sensory sensitivity and phobias [20, 21] during medical procedures with children who have ASD [22, 23]. Early work focused on using these strategies to collect neuroimaging data (MRI, EEG) in young children with and without developmental disabilities [24, 25], and more recently, comprehensive behavioral protocols have integrated ABA principles to collect neuroimaging data in children who have ASD. Nordahl et al. [26] developed a behavioral protocol grounded in ABA principles and utilizing the support of board-certified behavior analysts (BCBAs) to obtain awake MRI data (structural T1-weighted and diffusion-weighted images) in children with ASD 9 to 13 years with a broad range of intellectual ability (ranging from intellectual disability to age-appropriate). The protocol employed mock scan training to teach participants to lie still during the MRI exam with criteria established before continuing to the “real” MRI. All participants transitioned from mock to real MRI scans, and use of the protocol resulted in a success rate of 100% for T1-weighted images and 94% for diffusion-weighted images. While the majority of participants had successful image collection during the first or second attempt, up to five scan attempts were allowed. Although the success rate in this study was high, this study underscores the extensive time and resources needed to obtain high success rates (e.g., ability to attempt an MRI exam up to five times).

Subsequent protocols have built on these approaches to collect functional MRI data in youth who have ASD and low verbal and cognitive performance [27] as well as MRI and PET data in autistic adults with a range of IQ [28]. Gabrielsen et al. [27] incorporated anxiety reduction techniques, the use of noise-canceling headphones, and viewing “relaxing” visual images (the Inscapes paradigm) during the exam [29]. A scan success rate of ~ 80% was reported. Of note, however, scanning was not attempted with individuals with known difficulty with experiences such as dentist visits or haircuts, considered a proxy for their ability to hold still during the MRI scan. Smith et al. [28] incorporated at-home training with review of videos that familiarized the adult participants with the PET-MRI scanning procedures. All participants completed one training session, and 18 of 19 participants completed the entire protocol (scan success rate ~ 95%). Up to four attempts were required.

Similar behavioral interventions and protocols have been established for EEG (e.g., [15, 30–33]), though in general the demands on the child are lower and less “daunting” than MRI (e.g., the absence of a large and loud machine, less risk of claustrophobic experience, can be completed while sitting in a regular chair). Although more accessible for a wider range of individuals with cognitive or language limitations, EEG has limited spatial resolution and thus less accuracy for spatial localization [34]. More recently, functional near-infrared spectroscopy (fNIRS) has been suggested as an approach to measuring functional brain activity in ASD via measuring changes in hemodynamic responses (for review see [35, 36]). Although fNIRS has less susceptibility to the impact of motion and requires fewer trials than EEG, fNIRS sacrifices the capabilities of temporal resolution and/or spectral response characterization available to electrophysiology. fNIRS also suffers from poor depth resolution given properties inherent to scalp-mounted optodes, with fNIRS signals strongly biased towards the outermost 10–15 mm of intracranial space [37].

Compared to fMRI, EEG, and fNIRS, magnetoencephalography (MEG) offers advantages to studying brain neural activity, including providing excellent temporal and good spatial information [38]. The nature of the MEG technology also reduces some of the demands placed on an individual during the scan—participants can be in either a supine or seated position, there are no loud noises associated with MEG, and sensory demands are reduced due to the need for only a few sensors to be placed on the head [39].

Our laboratory has leveraged MEG to examine endogenous auditory cortex activity as well as early indicators of auditory language processing via the assessment of brain responses to tones and speech elements, such as vowels. Our research has documented auditory latency delays for children on the autism spectrum (e.g., [40]). These studies have also shown that the brain responses to auditory stimuli are associated with IQ and language ability in children with ASD without cognitive impairment [41, 42]. This finding, replicated across samples [43–46] and into adulthood [47], has, however, generally not been examined in children with cognitive impairment or significant language impairment (i.e., children who are MVNV). To observe MEG indices of auditory processing in children with ASD who have limited or no verbal speech and often co-occurring intellectual disability, an integrated clinical and technical protocol similar to those developed for MRI is needed with appropriate adaptations for MEG. There are unique features and procedures with MEG that need to be accounted for in an adapted protocol (e.g., the digitization process to map head shape, preparing for placement of the coils on the participant’s face). While families may have experience with the MRI and information about MRI can be easily accessed, they are less likely to be familiar with MEG procedures. As such there is a need to provide families with more specific guidance for home-based preparation than provided in previous protocols (e.g., practice plans incorporating pictures of MEG visit components with clear steps for families to follow, explanations for why each step in the process matters). Finally, we aimed to maximize a continuous “task analysis” throughout the protocol, leveraging the ABA concept of breaking down a skill or activity into smaller parts. As described below in the protocol description, this involved segmenting the MEG visit into smaller steps to identify a child’s possible challenge points during the screening process, providing targeted home-based practice plans for those particular challenge points, and then shaping and differentially reinforcing the child’s behavior during the visit as the team approached those challenge points.

To develop this protocol, we leveraged our key project associated with the NICHD-funded institutional Intellectual and Developmental Disabilities Research Center (IDDRC). The project was designed to fill the gap in understanding auditory processing in children with ASD who have developed little or no speech by the school-age years (8–12 years). This sample allowed for comparison to historically collected auditory response data in verbal, school-age (8–12 years) children with ASD and children with neurotypical development. Data collection for the larger project is ongoing and will also include a sample of age- and nonverbal IQ-matched children with intellectual and developmental disorders but without ASD.

Overall, the goal was to develop a protocol that would (1) broaden the inclusion of individuals with varying intellectual and language abilities in neuroimaging research and (2) improve data quality across age and performance range. In this methods paper, we present our clinical and technical protocol, MEG-PLAN (*MEG P*rotocol for *L*ow-language/cognitive *A*bility *N*euroimaging). After developing the protocol, we tested the feasibility of MEG-PLAN. Outcome measurements included (1) scan time/visit length, (2) scan success rate for both acquirable and evaluable data, and (3) data quality and reliability (i.e., test-retest reliability). Auditory neural response data from the pure-tone MEG paradigm (latency and amplitude) employed in this study is briefly summarized in the results section below, but is presented comprehensively in Roberts et al. [48].

## Methods

### Procedures

Study participation occurred across 2–3 visits to collect characterization and diagnostic information and MEG data. Characterization visits and MEG visits were each scheduled for at least 3 h to allow for flexibility in rapport building and MEG-PLAN preparation, desensitization and habituation procedures. Of note, if MEG data were not acquired during an initial MEG visit, a second visit was offered.

### Participants

Fifty participants with ASD were recruited and evaluated at phenotyping visits. Twelve children were excluded prior to the MEG imaging visit; eight did not meet eligibility criteria (i.e., scoring under thresholds on ASD diagnostic tools, deemed not minimally verbal, or head circumference too large for the MEG helmet) and four did not schedule MEG imaging visits (i.e., parent opted to not proceed to the MEG visit due to believing the child would struggle and be unsuccessful; parent unable to arrange MEG visit due to scheduling conflicts).

Data are reported for 38 MVNV children who have ASD (30 male, 8 female; mean age = 10.1 years, SD 1.4, range 8.2–12.7). All participants were assessed to obtain nonverbal IQ, to confirm that the participant was minimally verbal or nonverbal and to verify diagnostic criteria for ASD. Nonverbal IQ was obtained using the Leiter International Performance Scale, Third Edition [49]. For participants who had difficulty completing tasks on the Leiter-3 and could not reach basal levels of performance, developmental level (quotient) of nonverbal ability was estimated using the Mullen Scales of Early Learning [50] Visual Reception scale. Developmental quotients allow for an estimate of a child’s ability based on their own developmental level and chronological age rather than on standardized norms derived from same-age peers. Developmental quotients are calculated as developmental age/chronological age × 100. Developmental quotients are also presented for the Leiter-3 to allow for comparison with the Mullen Scales of Early Learning. Receptive and expressive language abilities were assessed using the Peabody Picture Vocabulary Test, Fourth Edition [51], and Expressive One-Word Picture Vocabulary Test—Fourth Edition [52]. MVNV status was operationalized as fewer than 30 words/phrases used functionally and spontaneously; status was confirmed with a natural language sample task. Diagnostic confirmation included the Autism Diagnostic Observation Schedule, Second Edition (ADOS-2) [53], and parent report on the Social Communication Questionnaire [54]. All participants were administered Module 1. In addition, four participants did not complete an ADOS-2 due to fatigue or distress during the evaluation visit. In these cases, diagnosis was confirmed with parent report on the SCQ and review of diagnostic records. To capture adaptive behavior skills and everyday functioning, parents/caregivers completed the Vineland Adaptive Behavior Scales, Third Edition (Parent/Caregiver Form) [55]. See Table 1 for participant demographics.

**Table 1:** Participant demographics—children who have autism spectrum disorder and are minimally verbal or nonverbal

	***N***	**Developmental quotient**		**Standard score**	
		***M*** **(SD)**	**Range**	***M*** **(SD)**	**Range**
Nonverbal IQ					
Leiter-3	34	46 (17)^a^	24–96	57 (15)	32–87
Mullen	3	23 (5)	17–26	--	--
		**Raw score**		**Standard score**	
		***M*** **(SD)**	**Range**	***M*** **(SD)**	**Range**
Receptive vocabulary	36	39 (20)	4–90	33 (13)	20–74
Expressive vocabulary	36	26 (23)^b^	0–90	56 (7)	54–86
Adaptive Behavior	38	--	--	52 (10)	34–70
Autism Diagnostic Observation Schedule, 2nd Ed (Calibrated Severity Score)	34	7 (1)	4–10		
Social Communication Questionnaire (Total Score)	38	26 (5)	14–33		

^a^*N* = 33, one participant had data shared from a recent Leiter-3 administration and raw scores were not available, thus developmental quotient could not be derived

^b^For the expressive vocabulary assessment 10 participants were nonverbal with raw score *=* 0. The floor standard score is < 55; a standard score of 54 was universally included for these cases, but is likely artificially inflating the standard score mean value

*Nonverbal IQ* Leiter International Performance Scale, 3rd Edition (Leiter-3), Mullen Scales of Early Learning (Mullen); *Receptive Vocabulary* Peabody Picture Vocabulary Test, 4th Edition (PPVT-4); *Expressive Vocabulary* Expressive One-Word Picture Vocabulary Test, 4th Edition (EOWPVT-4); *Adaptive Behavior* Vineland Adaptive Behavior Scales, 3rd Edition, Parent/Caregiver Form Adaptive Behavior Composite (Vineland-3 ABC Composite)

When *N* < 38, missing data reflects participant fatigue or distress that resulted in an abbreviated research visit

### MEG-PLAN

**Fig. 1:**
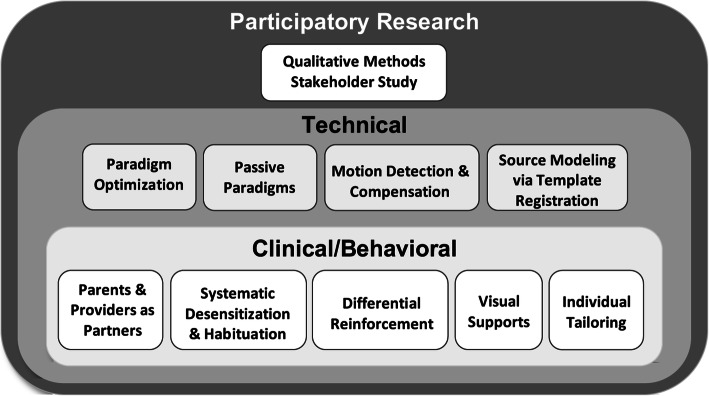
MEG Protocol for Low-language/cognitive Ability Neuroimaging (MEG-PLAN)

MEG-PLAN (MEG *P*rotocol for *L*ow-language/cognitive *A*bility *N*euroimaging) was developed using stakeholder feedback from parents and caregivers (parents hereto forward) of MVNV autistic children and providers who work with them. We conducted walking interviews or “go-alongs” [56, 57] with parents and providers to gain an enriched and deeper understanding of what our research study experience would be like for children and families, and how we could improve the process. Common themes in the feedback included making the laboratory space as child-friendly as possible (i.e., minimizing the machine and technology “presence”) and encouraging our team to consider that a social story/storybook and video may have variable utility depending on the child. Stakeholder feedback also suggested that we leverage the child’s ability to watch a movie (with no audio track) during the MEG scan and that starting the movie during the scan preparatory activities could help capture the child’s attention and focus, allowing for a more seamless transition into the MEG machine. Anecdotally, although not uniformly effective, this approach was often helpful and utilized by study staff. To develop MEG-PLAN, we integrated (1) the stakeholder parent/provider feedback, (2) previously described protocols [15, 26, 27], and (3) information provided via close consultation with our interdisciplinary team, including physicists, neuroscientists, clinical psychologists, engineers, MEG technologists, and behavior analysts. The resulting MEG-PLAN is a multipronged protocol, comprising clinical/behavioral and technical strategies. See Fig. 1.

### Clinical/behavioral components

#### Parents and providers as partners

Parents are included at each step of the protocol and provide crucial input. Providers, such as in-home therapists or school para-professionals/one-to-one aides working closely with the child, also often participate as active partners at the MEG visit. Indeed, in some cases, parents indicate that the provider is best aligned with the child in terms of implementing behavioral management strategies and supporting cooperation to meet task demands. Prior to the phenotyping and imaging visits, parents (and providers when pertinent) are sent a video that describes the MEG process [58]. Parents then complete an intake interview with a behavioral specialist to identify sensory challenges related to MEG techniques and provide information about the participant regarding challenging behaviors, preferred reinforcers, and behavioral strategies/plans (see comments regarding the crucial role of this behavioral specialist in the “Implementation of MEG-PLAN” section). Information specific to the MEG technology is also collected to prepare for the visit (e.g., whether the child would be most comfortable with the MEG in an upright chair position or supine laying down position). Supplementary data shares the details of this “MEG-PLAN Pre-Visit Intake Interview” (see Additional file 1).

Parents and providers are involved during the phenotyping and imaging visits to provide additional behavioral support as needed. During the phenotyping visit, parents or providers sit in the room if there are any concerns for elopement, and they provide input on effective behavioral strategies. Parents or providers are also in the room during MEG data collection to provide additional support and input (e.g., identify early signs of escalation). They also often assist in modeling behaviors (e.g., allowing the team to place the MEG head position coils on their own face prior to placing the coils on the child’s face), helping the team to understand the child’s communication attempts, and in reinforcing appropriate behaviors.

#### Systematic desensitization and habituation

To support acclimation to the MEG environment, decrease anxiety, and increase comfort in participants and their parents, systematic desensitization, and habituation are used. Systematic desensitization is a technique used to treat anxieties and phobias via gradually exposing an individual to an unwanted stimulus [59, 60]. Habituation occurs when there is a decrease in the magnitude or even a lack of a response after repeated exposure to a stimulus [61].

Systematic desensitization and habituation are implemented for all participants during the MEG visit. The behavior specialist introduces each step in the MEG process to the participant verbally and visually, and models the procedures for that step using relevant materials (e.g., first demonstrates how to sit in the MEG chair and then asks the child to sit in the MEG chair). The MEG set-up procedure is broken down into discrete steps using a task analysis, and materials are then introduced to the participant step-by-step. For participants who demonstrate sensory aversions (i.e., withdrawing from the materials), the behavior specialist introduces the materials more gradually, first using their finger to demonstrate where the materials will be placed, then placing the materials on the participant’s hand or allowing the participant to feel the materials. The behavior specialist uses hand-over-hand prompting to give the participant a sense of control over the placement of the materials, and if needed, introduces the materials briefly (i.e., for 5 seconds) before gradually increasing the amount of time exposed to the materials.

If during the intake interview parents identify particular sensory aversions, or the participant demonstrates sensory aversions during the phenotyping assessment visit, a “practice plan” is developed and provided to the family. A practice plan is a step-by-step handout showing/describing the materials and how long this portion of the procedure takes and provides additional information for families to practice at home prior to the imaging visit. For example, if a parent identified keeping head position coils on the participant’s face as a potential challenge, the parent is provided a practice plan for putting coils on the participant’s face, along with some paper tape and plastic string (i.e., “wires”) to use when practicing at home. The plan also notes the locations on the face where the coils are placed and includes strategies for gradually introducing the coils to the participant. For example, it may be recommended to first try holding one coil to the participant’s face for a few seconds, and then gradually increasing the amount of time the participant tolerates the coil on their face before taping the coil onto the participant’s face, and then finally introducing additional coils.

**Fig. 2:**
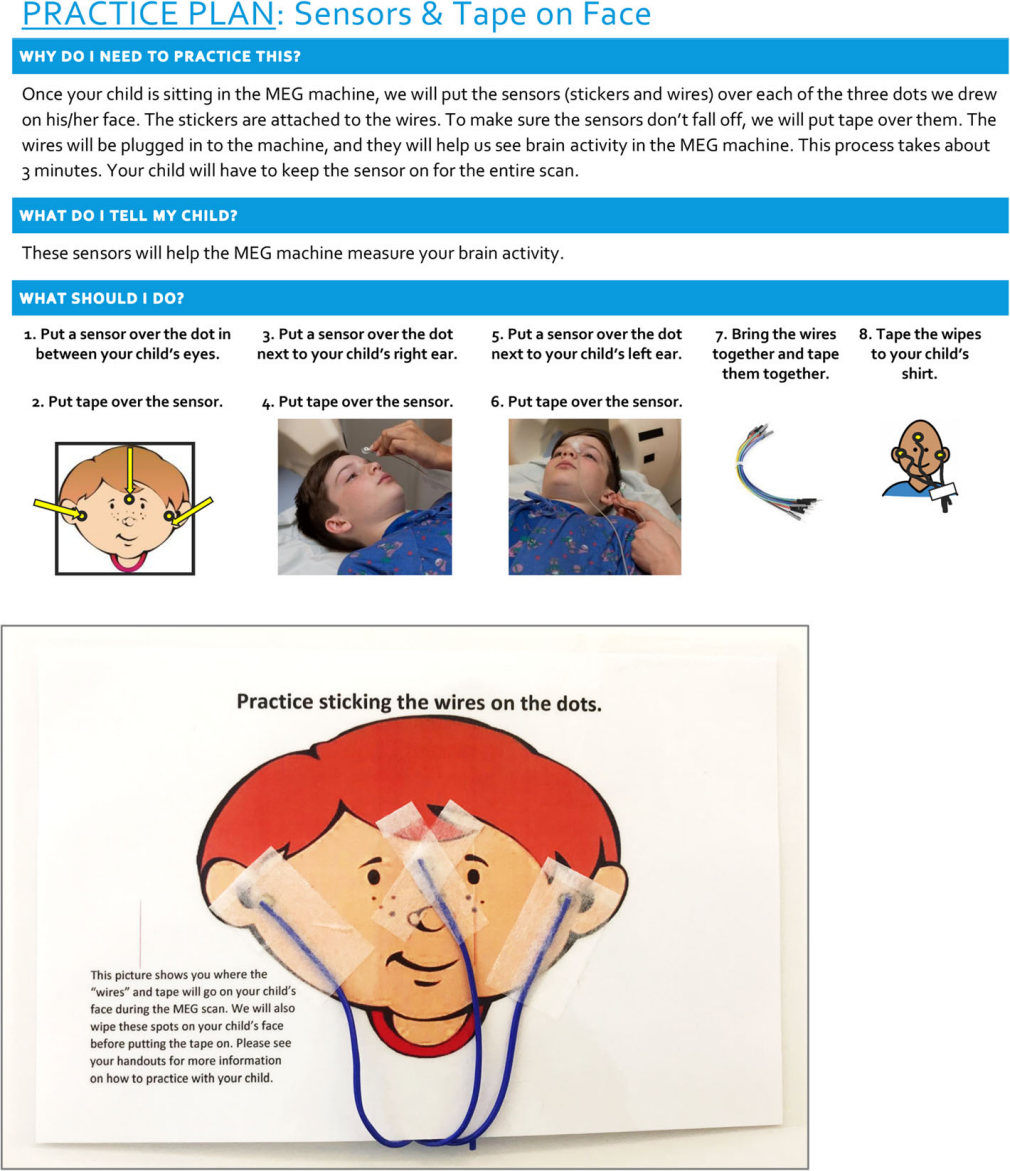
Sample practice plan and accompanying materials to support family preparation for MEG visit. **a** (top) Sample practice plan provided to families prior to MEG visit. Plan describes the background justification for why the procedures are necessary, how to describe the procedure to the child, and step-by-step instructions for practice and desensitization. **b** (bottom) Sample materials provided to parents to support practice plan. In this case, a laminated picture of a face is provided with practice wires (waxed/plastic string) and samples of the paper tape used to affix the coils (“wires”) to the face. Additional pieces of paper tape are provided to allow for additional practice and desensitization

Practice plans are accompanied by a “General Tips” handout that suggests additional strategies to be incorporated into the practice plans (e.g., a timer or counting, keeping practice sessions short and fun, incorporating existing reward systems during practice times, reminding parents that some children may need multiple sessions to master one step before moving to the next). See Fig. 2 for a sample practice plan and accompanying materials. Finally, and of note, this process provides ample time for desensitization procedures in a familiar and comfortable environment without the family or team feeling pressured by time constraints.

#### Differential reinforcement

Differential reinforcement involves implementation of procedures to increase desired behaviors while extinguishing, or decreasing, undesired behaviors [62]. Differential reinforcement is used to increase behaviors that are compatible with scanning, such as sitting in the MEG chair. These behaviors are shaped by breaking the behavior into successive approximations which require the participant’s responses to more closely resemble the targeted behavior in order to receive reinforcement [63]. During the intake interview, families are asked to provide a list of preferred objects and activities to be used as reinforcers. The team uses a first-then board or language to communicate behavioral expectations and the activity or object that the participant would earn for following through with the behavior (e.g., “First sit, then movie”). Participants are granted access to the objects for approximations of a behavior that are compatible with scanning. For example, if a participant avoids sitting in the chair but is motivated by watching a movie, the team gradually shapes the participant’s approach to the chair using differential reinforcement and shaping. To this end, the team might play the preferred video as the participant walks closer to the chair but would then pause the video if the participant attempts to walk away. Once the participant is near the chair, criteria for playing the video is raised, such that the participant has to sit in the chair for a brief period of time, and gradually sit in the chair for longer periods of time, in order to continue watching the video. Differential reinforcement is used throughout the scan to acknowledge when the participant is sitting still and quiet. Additional reinforcement strategies, such as token economy systems, are also used.

#### Visual supports

**Fig. 3:**
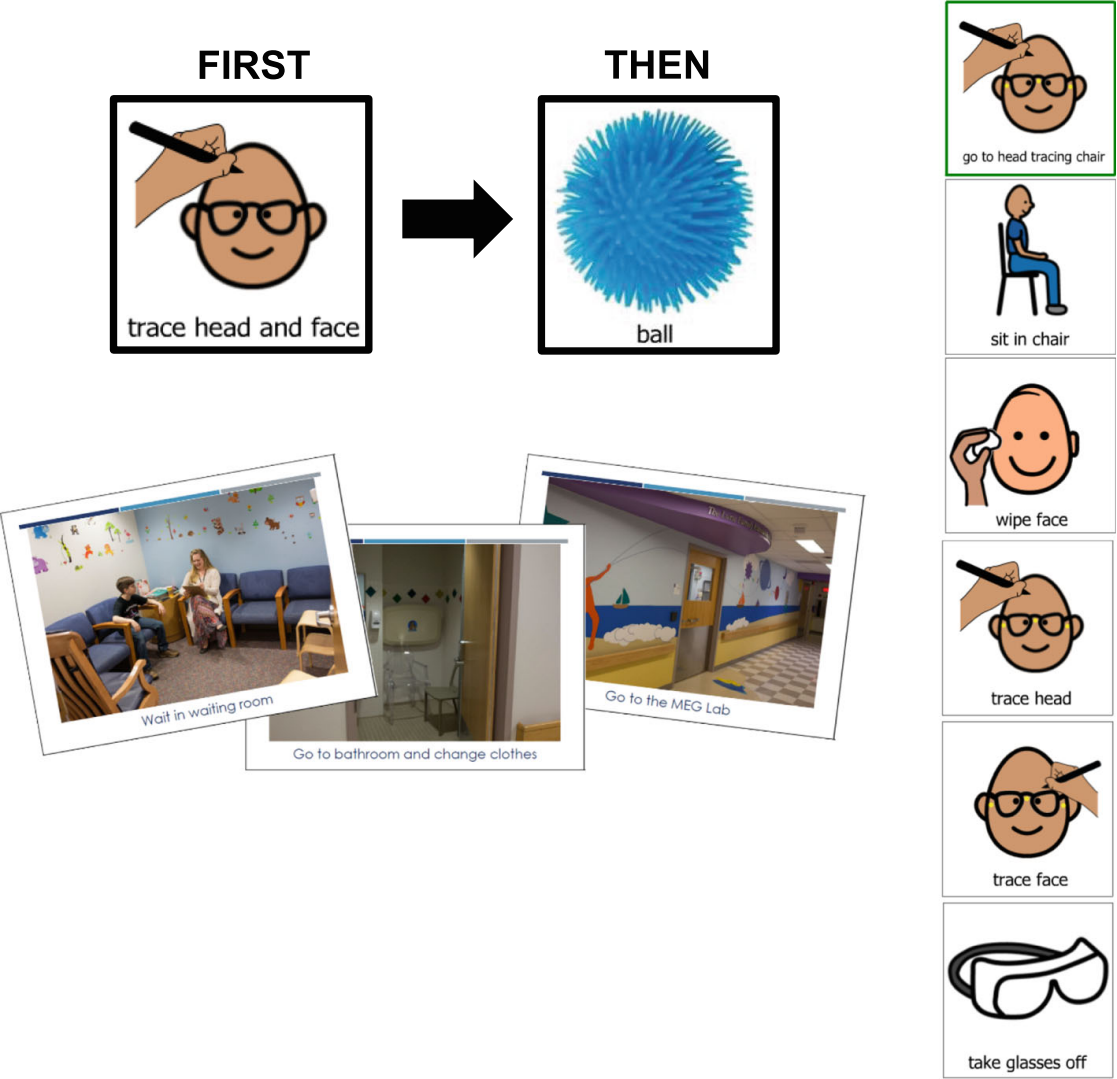
Sample visual supports used in MEG-PLAN. **a** (top left) Sample first-then board used to communicate behavioral expectations and/or the next step in the schedule, followed by the reinforcer the participant would earn for completion of the step. **b** (bottom left) Sample pages from a picture book. This book was reviewed with the participant and parent to familiarize them with the MEG process. It was also sometimes used as a visual schedule for each step in the MEG visit process. **c** (right side) Sample visual schedule that breaks down behaviors, such as digitizing, into smaller, discrete steps

Visual supports are pictures or other visual tools (e.g., written schedule) used to aid communication in children with language impairments. Visual supports help a child understand and follow spoken instructions, and can help children understand what to expect in unfamiliar situations. For MEG-PLAN, visual supports are provided via multiple modalities and for multiple purposes. First, a video demonstrating the MEG data collection process is sent to parents (see YouTube link in “Parents and Providers as Partners” section). Parents are encouraged to watch the video with the child. Next, a picture storybook outlining each step of the visit is shown to the participant at the beginning of the visit. The pictures from the book are then used as a visual picture schedule during the visit to break the process into smaller, more focused steps. Throughout the MEG visit, visuals are also used to communicate rules (e.g., staying quiet, hands down) and label spaces in the environment to facilitate transitions (e.g., bathroom, digitizing room) and to support motivation and differential reinforcement strategies (e.g., first-then board with preferred toy available at a break or picture of the prize box at the end of the visit). See Fig. 3 for a sampling of the visual supports used in MEG-PLAN.

#### Individual tailoring

MEG-PLAN is individualized for each participant based on his or her specific needs. Motivators and behavioral strategies are tailored based on information collected prior to the MEG visit. If participants have interests in particular objects or topics, such as elevators or Thomas the Train, the team prepares video playlists, visual supports (e.g., token boards), and toys or activities based on that interest. The team also implements verbal cues (e.g., “Eyes on me”, “Ready hands”) or behavioral strategies consistent with what the participant is familiar with from the home, school, or treatment environment in order to reduce the need to introduce and teach new terminology. The participant is allowed to make choices whenever possible to provide a sense of control (e.g., which fiducial marker or “dot” to draw on the face first).

#### Technical components

In addition to the clinical and behavioral strategies to support participants through the duration of the MEG visit, MEG-PLAN includes a number of technical considerations to tailor the MEG data collection to the needs of this population. Chief among these is the use of *Passive Paradigms* to support children who find it difficult to cooperate with task demands and to maintain consistent attention and vigilance to task stimuli. The MEG tasks included for the study with the sample described in this paper relied on 100% passive presentation paradigms of simple auditory stimuli—tones, vowel sounds, and word/word-like stimuli. Participants are not asked to respond or attend to the stimuli but instead instructed to focus their attention to a movie or video of their choice (played without sound). Obligate brain responses are collected. Similarly, *Paradigm Optimization* is considered to minimize overall scan length and incorporate opportunities for breaks. Additionally, consideration is paid to the way stimuli are presented; for example, the auditory stimuli used in these tasks are presented using a directional flat panel speaker as opposed to in-ear earbuds or over-ear headphones, to be more easily tolerated by participants with sensory aversions. Data analysis procedures are also adjusted to better accommodate the population being studied. *Motion Detection and Compensation* (continuous motion detection) during the MEG scan is achieved via the use of three active coils, placed at anatomic landmarks. The locations of these coils are identified at a millisecond time scale and can be subsequently used to detect, gate, covary, or otherwise compensate for participant head motion, decreasing the need for participants to remain extremely still for the entire MEG scan. Finally, the use of age-matched *MRI templates for source modeling* eliminates the need for a structural MRI exam while preserving the fidelity of the reconstructed signals [64], as well as the signal averaging/combination benefit intrinsic to source modeling from multiple sensors.

### Implementation of MEG-PLAN

MEG-PLAN is implemented in three phases:

1. *Assessment*. Behavioral assessment and phenotyping visit summary
2. *Plan and preparation*. MEG clinical support and home practice and preparation
3. *MEG visit*. MEG clinical components and MEG technical components

**Fig. 4:**
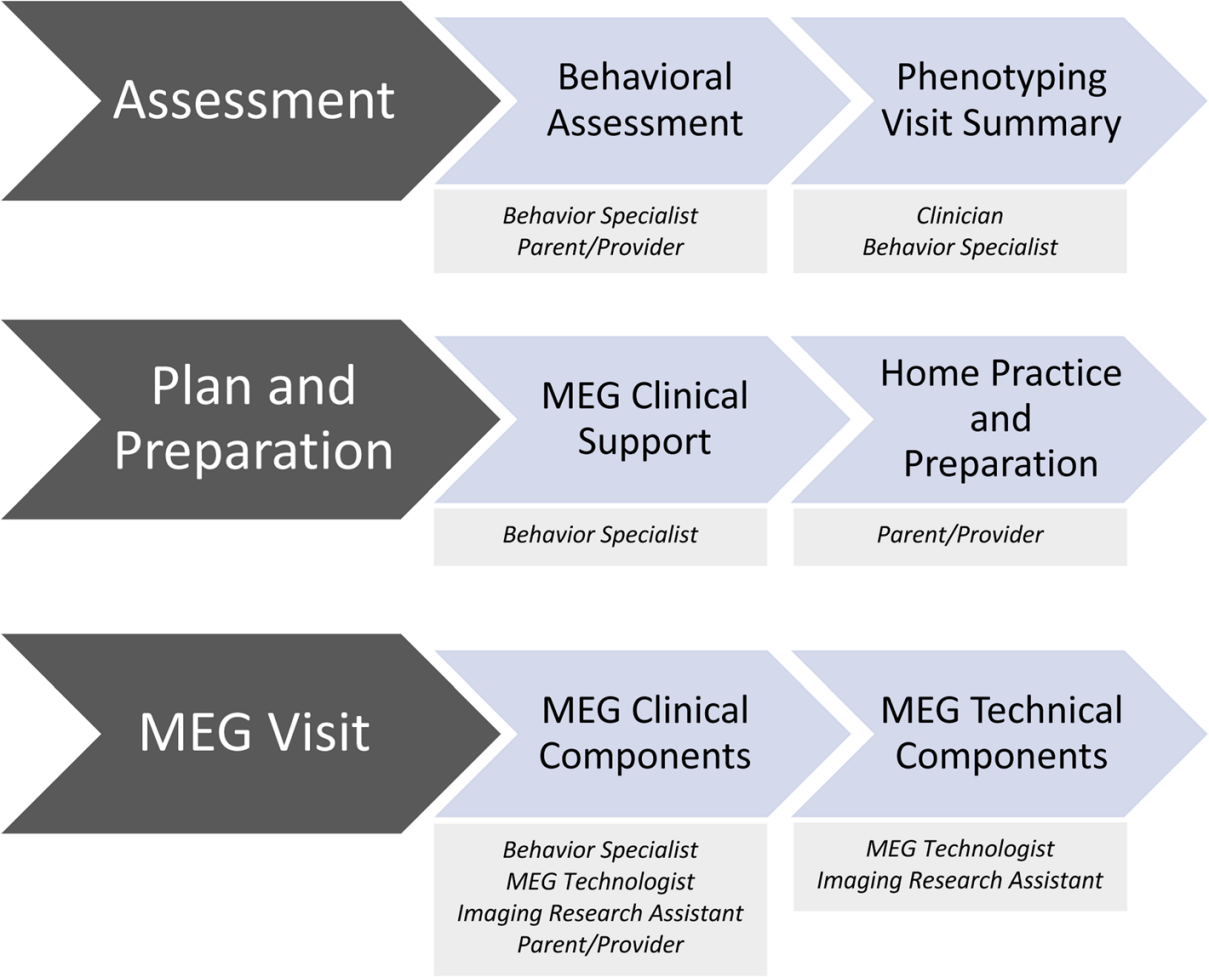
Three phases of MEG-PLAN implementation and the interdisciplinary team members needed for each phase

The integration of an interdisciplinary team (behavior specialist, clinician, MEG technologist, neuroimaging research assistant) and the parents and/or providers involved in MEG visit preparation is crucial to MEG-PLAN implementation success (see Fig. 4; also see Additional file 2, “Three Phases of MEG-PLAN Implementation” for a comprehensive description of how each phase is implemented). MEG-PLAN is designed to be individualized to each child’s needs based on the information collected in the assessment phase; it is not intended to be fully manualized. MEG-PLAN could be thought of as a “modular” protocol with a set of components that can be combined to meet each child’s needs for the imaging visit. It is recommended to put great effort into including a behavior specialist on the imaging team. This specialist should have a background in the application of individualized behavioral strategies. A board-certified behavior analyst (BCBA) can serve as an excellent consultant or per diem team member in this instance, although others with similar background and training can also fill this role, thus our reference to behavior specialist more broadly. While some MEG-PLAN strategies are used universally, slight variations in implementation are made based on the child’s familiarity with previously used strategies. For example, whereas one child can respond to verbal presentation of “First sit, then movie,” another child may need a more structured “first-then” board with pictures. Expert consultation in these types of personalized adjustments is extremely valuable. Moreover, the goal of continuity and task analysis of challenge points throughout the process is best implemented by the behavior specialist, who can keep the overarching themes for a particular child at the forefront across the assessment, plan/preparation, and MEG visit phases.

### MEG paradigm and data collection

MEG visits were scheduled for 3 h to allow for ample time to implement MEG-PLAN and collect MEG data. As part of the larger IDDRC project, three paradigms were attempted based on child engagement, attention, and fatigue. Paradigm length ranged from 4 to 15 min, with a total possible of 45 min of direct scan time. Of note, the pure-tone auditory task described below was always presented first. After successful completion of this auditory task, additional paradigms were attempted. Breaks were taken in-between paradigms as needed.

Data included in this paper reflects a 14-min passive auditory task with sinusoidal tones (300 ms duration; 10 ms ramps) presented with a pseudo-randomized 600–2000 ms inter-trial interval using a freefield loudspeaker (and thus binaurally) approximately 2 m from the participant at 85 dB SPL using Eprime v1.1 experimental software (Psychology Software Tools Inc., Pittsburgh, PA). Stimuli were presented across 520 trials, a number larger than most paradigms in the literature. This design anticipated excluded trials and potential data loss due to head (or body) movement. MEG data were obtained in a magnetically shielded room using a 275-channel whole-cortex CTF magnetometer (CTF MEG, Coquitlam, Canada). Three head position indicator coils were attached to the scalp to provide continuous specification of the position and orientation of the MEG sensors relative to the head. If tolerated, foam wedges were inserted between the side of the participant’s head and the inside of the MEG dewar to increase participant comfort and ensure that the head remained in the same place in the dewar across recording sessions. To minimize fatigue and encourage an awake state, participants viewed a movie (of their choice, but without sound) projected on to a screen positioned at a comfortable viewing distance. A member of the clinical team and the parent remained in the room with the participant during data collection to ease anxiety and continue needed behavioral supports, communicate with the MEG technologists about breaks, and prompt participants to remain quiet or still.

### MEG data analysis

Epochs 100 ms pre-stimulus to 500 ms post-stimulus were defined from the continuous recording. Epochs with artifacts were rejected by amplitude and gradient criteria (amplitude > 1200 fT/cm, gradients > 800 fT/cm/sample). Noncontaminated epochs were averaged and a 1 (12 dB/octave zero-phase) to 55 Hz (48 dB/octave, zero-phase) band-pass filter was applied.

Using all 275 channels of MEG data, determination of the strength and latency of M50 responses in the left and right superior temporal gyrus (STG) was accomplished by applying a standard source model to transform each individual’s raw MEG surface activity into brain space (MEG data co-registered to an age-matched MRI template: https://jerlab.sc.edu/projects/neurodevelopmental-mri-database/) using a model with multiple sources [65, 66]. In particular, the standard source model applied to each subject was constructed by including left and right STG dipole sources (placed at left and right Heschl’s gyrus) [66, 67]. This source model served as a source montage for the raw MEG [65, 66]. As such, the MEG sensor data were transformed from channel space into brain source space where the visualized waveforms are the modeled source activities. This spatial filter disentangles the source activities of the different brain regions that overlap at the sensor level. Of note, although the latency of the ~ 50 STG responses were obtained using a dipole source placed at a standard location, in each subject the left- and right-hemisphere dipoles were oriented at the maximum of the individual M50 response. As such, orientation of the standard STG sources was optimized in each subject.

### Outcome measures

MEG-PLAN feasibility was evaluated in three ways. First, *scan time and visit length* confirmed whether MEG-PLAN could be implemented and data collected within the time slot established for the MEG visit (i.e., 3 h). For participants with acquirable data, scan time was operationalized as time spent with an active paradigm running, inclusive of breaks taken in between paradigms to understand the length of time required to complete the full scan protocols. Second, *scan success rate* was evaluated for both acquirable and evaluable/analyzable data. For the purposes of this paper, acquirable data is data collected when the MEG machine is turned on and a paradigm administered, without consideration of data quality. Evaluable data is the higher threshold of trials free of artifact that can be included in data analysis. We have chosen to differentiate and include both acquirable and evaluable scan success rates as paradigm features can impact whether data quality is evaluable and analyzable (e.g., length of task, order of task within scan visit). We believe reaching the point of initiating a paradigm in a neuroimaging study is an important milestone to consider as a key stepping stone to collection of quality, evaluable data. Finally, auditory neural response data from the MEG paradigm (pure-tone auditory task) were evaluated for data quality (evaluable trial counts) and reliability (test-retest reliability). A preliminary examination of the pure-tone auditory latency and amplitude findings are briefly summarized, based on published data from a subset of this ongoing study [48].

## Results

### MEG-PLAN feasibility

#### MEG scan visit length

Median scan time was 45 min (range 4 to 63 min), with approximately half of the participants taking no breaks and the other half taking one or two breaks; one participant took three breaks. Preparation time in the MEG scanner (i.e., desensitization and habituation procedures) was tracked for a subset of participants (*n* = 12); preparation time was ~ 10–30 min for the majority of participants (*n* = 9), with two participants preparing in less than 10 min, and one participant requiring over 1 h. These data (from participants with acquirable data) demonstrate that MEG-PLAN can be implemented and auditory neural responses collected within the 3 h window allotted for these study visits.

#### Scan success rate

Of the 38 participants who attended an MEG visit, acquirable data were obtained from 26 participants on the first MEG visit (scan 1). In all cases, when data could not be obtained at scan 1 (e.g., child could not keep head localization coils on face, child could not stay in the MEG helmet), a second MEG visit (scan 2) was offered. Five children completed a scan 2 visit, with two yielding acquirable data. Seven children did not return for scan 2 due to logistical challenges (e.g., notable distance between home and medical center, parent unable to take time off from employment). Taken together, MEG-PLAN yielded 74% scan success rate for acquirable data (*N* = 28 of 38 participants). When considering evaluable/analyzable data, for the pure-tone auditory task, 71% of those with acquired data (*N* = 20 of 28 participants) showed data quality acceptable for evaluation and analysis.

Table 2 characterizes the data based on acquirable or evaluable/analyzable “group” status. Exploratory analyses (with no corrections for multiple comparisons) suggested trends for group differences in nonverbal IQ, *F* (2, 31) = 2.7, *p* = .08, *η*_*p*_^2^ = 0.15, and adaptive behavior skills, *F* (2, 35) = 2.6, *p* = 0.09, *η*_*p*_^2^ = 0.13. Pairwise post hoc tests indicated lower nonverbal IQ in the non-acquirable group than the acquirable/non-evaluable group (*p =* 0.03), and lower adaptive behavior scores in the non-acquirable group than the evaluable group (*p =* 0.03). All other comparisons were nonsignificant (*p*s > 0.29).

**Table 2:** Participant Demographics for Evaluable, Acquirable/Non-evaluable, and Non-acquirable Groups

	Evaluable	Acquirable/non-evaluable	Non-acquirable
	***M*** (SD) range	***M*** (SD) range	***M*** (SD) Range
*N*	20	8	10
Age (years)	10.2 (1.5) 8.2–12.7	9.5 (0.8) 8.4–11	10.2 (1.3) 8.5–12.3
Sex (*M*:*F*)	16:4	6:2	8:2
Nonverbal IQ (Leiter-3 Standard Score)	57 (13) 32–81	64 (17) 36–87	48 (14) 34–77
Receptive Vocabulary (PPVT-4 Raw Score)	41 (20) 15–90	44 (15) 24–65	29 (24) 4–78
Expressive Vocabulary (EOWPVT-4 Raw Score)	26 (20) 0–72	32 (28) 0–90	19 (24) 0–67
Adaptive Behavior (Vineland-3 ABC Standard Score)	54 (9) 34–70	53 (10) 39–68	46 (9) 35–58
Autism Diagnostic Observation Schedule, 2^nd^ Ed (Calibrated Severity Score)	6 (1) 4–9	7 (2) 4–9	7 (2) 6–10
Social Communication Questionnaire (Total Score)	26 (5) 19–33	28 (4) 22–33	24 (6) 14–32

#### Evaluable data quality and reliability

Evaluation of trial counts and data quality revealed a wide range of acceptable trials across the 20 participants with evaluable data (184–516, *M* = 392.60 ± 91.46, Median = 417.50). Of note is that although total trial count was low for several “outlier” participants, M50 responses were still observed in these participants and the remaining participants showed trial counts on par with those reported in historical studies within our laboratory (see [48]; mean trial count for verbal children who have ASD = 459.2, SD = 3.7). Of note, for the participants with evaluable/analyzable data (*N* = 20), trial count was not associated with any characterization variable (modest relationship for trial counts with age, *r* = − 0.23 and with nonverbal IQ, *r* = − .32; all other *r*s ≤ 0.09).

**Fig. 5:**
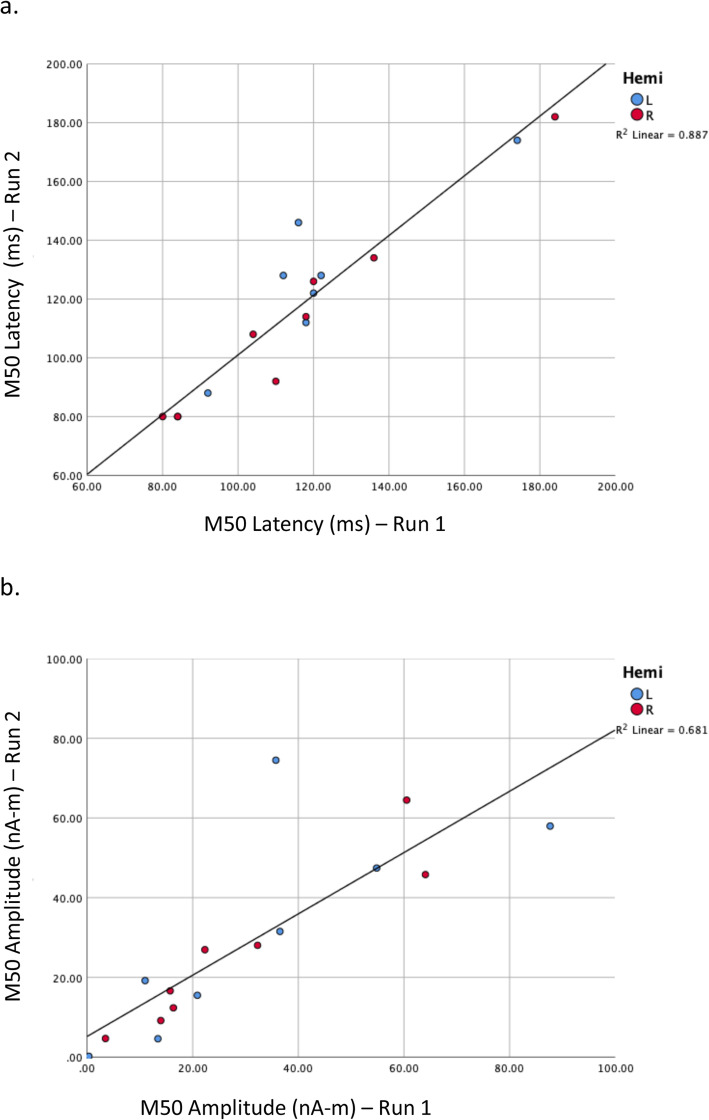
Test-retest of pure-tone (M50) latency and amplitude. Legend: Test-retest of pure-tone (M50) latency and amplitude determination in eight minimally verbal/nonverbal children on the autism spectrum confirms the feasibility of the MEG-PLAN approach as well as the reliability of latency measures

Given that, in many subjects, data quality was compromised due to significant movement and or other artifact (e.g., metal dental work or clenching muscles in face), the first set of participants with acquirable and evaluable data (*N* = 8) were scanned twice on the initial auditory task to evaluate test-retest reliability. High intraclass correlation coefficients values for M50 latency and, to a lesser extent, amplitude in both hemispheres (see Fig. 5), demonstrated reliable measurement of the M50 response even in the presence of significant movement and noise in some participants.

### Pure-tone auditory task findings

**Fig. 6:**
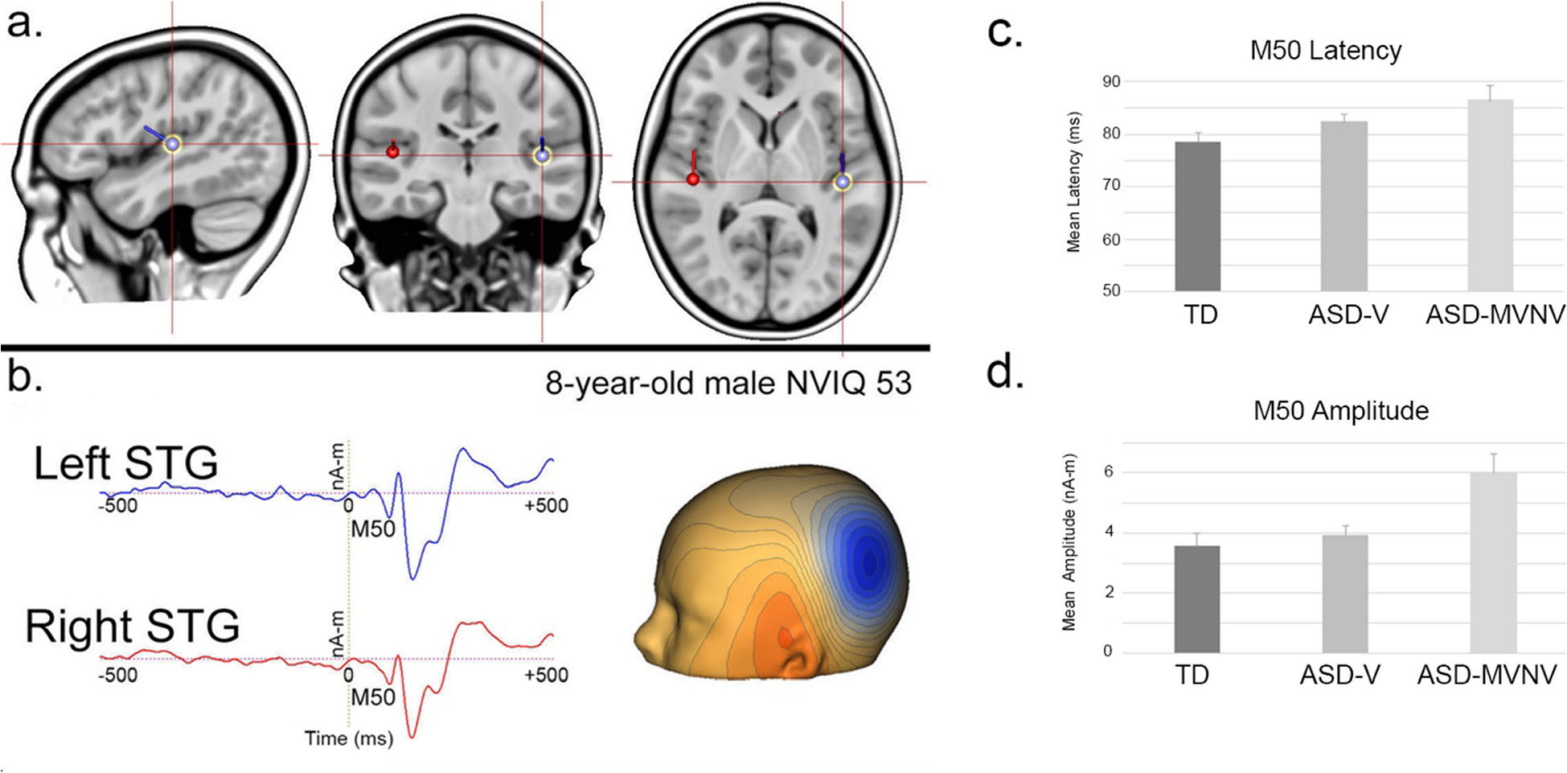
**a** Left and right superior temporal gyrus (STG) dipoles placed and left and right Heschl’s gyrus. **b** Left and right STG auditory source waveforms from an 8-year-old male. Left and right STG M50 responses are observed (left M50 field map shown). **c** Mean M50 latency values for each group with standard error bars. **d** Mean M50 amplitude values for each group with standard error bars

The focus of this paper is on the development and feasibility of the MEG-PLAN protocol. Figure 6a shows left and right superior temporal gyrus (STG) dipoles placed and left and right Heschl’s gyrus. Figure 6b shows left and right STG auditory source waveforms from an 8-year-old male. Left and right STG M50 responses are observed (left M50 field map shown). A subset of data (*N* = 16) leveraged for this study has been published and compared to historical samples from our laboratory. As presented in Roberts et al. [48], delayed M50 latency was observed in the children with ASD and MVNV language versus a group of typically developing children, and with a nonsignificant trend for children with ASD and MVNV language to show delayed M50 latency relative to verbal children with ASD. Regarding M50 amplitudes, the children with ASD and MVNV language appear to show stronger M50 responses than the typically developing children and verbal children with ASD (see Fig. 6c and d for latency and amplitude mean and standard deviation for the left and right hemisphere). The above findings, reported in full in Roberts et al. [48], showed differences in M50 measures between the two ASD groups, and thus provide one example of the need to include a range of individuals with ASD in neuroimaging studies in order to formally assess the similarity in brain activity between lower and higher functioning individuals with ASD.

## Discussion

Neuroimaging research conducted with individuals on the autism spectrum has historically excluded children who are nonverbal or have intellectual disability. Inclusion of individuals with lower cognitive and language abilities in neuroimaging research is critical to obtaining a more complete understanding of ASD and other associated disorders. MEG-PLAN builds on previously established MRI protocols to allow for collection of neurophysiological data with individuals typically not included in neuroscience research. Scan success for acquirable MEG data occurred at a rate of 76%, and evaluable/analyzable data at a rate of 71% of those acquired. There was high reliability of auditory pure-tone auditory latency and, to a slightly lesser extent, amplitude.

The high reliability of left and right auditory M50 latency demonstrates that these auditory encoding measures are validly attained. The high reliability is of note given that the MEG data from many children in this study had significant metal artifact from dental work (dental work such as fillings and caps is likely more common in MVNV and ID populations given that MVNV and ID populations have more difficulty maintaining good dental hygiene for a variety of reasons [67]). More generally, the present study demonstrated the feasibility of extending MEG recordings to a broader representation of the autism spectrum. It is anticipated that this will lead to more generalizable findings as well as a more detailed understanding of the neural correlates of ASD across a wide range of abilities.

Our scan success rate for acquirable data is in line with the rate reported by Gabrielsen et al. [27], who reported a ~ 80% fMRI success rate in their children (7–17 years) on spectrum with low verbal and cognitive performance. Nordahl et al. [26] reported higher success rates using mock scanning and motion sensor training procedures. There are several reasons why acquirable data were not obtained in 26% (*n* = 10) of the children in our sample. First, as in other studies, it is likely that some of these children would have had a successful second scan if attempted. As previously noted, for some, a second MEG visit was not possible given that the family lived too far away, the parent was not able to take time away from work an additional day, or the parent did not want the child to miss an additional day of school. For others, the lack of success at a first visit may have decreased the parents’ confidence and enthusiasm for trying a second time. These observations highlight the need to try to recruit participants who live close to the imaging center as well as the need to prepare parents for the possible need for an additional visit.

Preliminary analyses examined variables that might relate to scan success. Findings suggested that stronger nonverbal cognitive abilities and adaptive behavior skills *may* be associated with reaching the point of acquiring MEG data. Of note, these data should be interpreted with caution given the small sample size and no correction for multiple comparisons given the exploratory nature of these analyses. From a clinical perspective, the skills required for completing everyday life tasks (e.g., attending to and following directions, managing anxiety and regulating emotions, communicating for social interactions) are well aligned with the skills needed for scan success. Adaptive behavior skills have been shown to be dissociated from symptom severity in children who have ASD [68], and a similar pattern may be evident with scan success for acquirable data given the absence of associations with ASD characterization measures (i.e., ADOS-2 and SCQ). For participants with acquirable data, none of the variables included in present analyses differentiated those with or without evaluable data. There were also no clear associations with evaluable trial count, though there was between-subject variability in acceptable trial counts, suggesting heterogeneity even within the evaluable cohort. While this could be due to low sample size in this subgroup (*N* = 8), it will be important for future work to explore other participant characteristics (e.g., sustained attention skills, temperament, learning readiness) and aspects of the MEG-PLAN protocol that will identify those most likely to make the transition from acquirable to analyzable data.

It is important to note that it is our practice to move *all* children forward to an MEG visit unless we have concerns of unduly stressing the child (e.g., the child has a phobia of the hospital or imaging machines), or if there are concerns about the ability to keep the child and research team safe throughout the study (e.g., the child has high risks for aggression or elopement without a solid behavior support plan and family/personnel supports to manage these possible crises). This approach differs from other protocols where mock scanning procedures serve as a “gatekeeper” or threshold to moving to a full imaging visit. Although the opportunity for a mock scan visit is provided if we believe this might be helpful, families are often not enthusiastic about additional visits that are not the “real” scan. Note that, unlike many MRI studies, we did not use mock scan tolerance as a screen for study entry. As there is no commercially available (and realistic) MEG mock scanner, doubts would remain about the generalizability thereof. As such, the real MEG device is used for habituation, and we always record in case of success. This approach maximizes acquirable data opportunities but may exacerbate the difference between acquirable and analyzable data rates. And although there are costs associated with maximizing acquirable data (i.e., paying for scans that do not yield evaluable data), this allows for a more inclusive research approach. Thus, although our success rates may be lower than the success reported for MRI, our procedures are likely less exclusionary.

There are also differences between what procedures can be used in a laboratory located in an urban clinical (hospital) versus a non-clinical (psychology department or independent research center) environment. Laboratories in a clinical setting having greater restrictions regarding use of the neuroimaging devices and laboratory/waiting space, versus non-clinical labs where research teams have much greater autonomy, flexibility in scheduling and procedures, and greater access to MRI or MEG systems. In the case of the present study, the MEG machine and staff are housed within a large children’s hospital, with the MEG system used intensively for clinical practice (epilepsy exams) and research studies. MEG-PLAN reflects procedures that can be implemented successfully even within the restrictions of that environment. Although MEG is not as available as MRI or EEG, the MEG-PLAN procedures are generalizable to almost any brain imaging study. And although access to an on-staff certified behavioral analyst may be limited, behavior specialists implementing high-level applied behavior analysis services are valuable team members and are often available for contract work.

Initial MEG-PLAN feasibility and scan success data offers a promising foundation for future work in this area. Data collection in this first study offers a global perspective on implementation of the MEG-PLAN protocol. More fine-grained and comprehensive data collection in future studies will allow for evaluation of individual components of the protocol (e.g., parent report of preparation time with protocol materials, parent versus outside provider support during MEG visit). In addition, larger datasets will lay the groundwork for identifying possible predictors of maximal scan success. The combination of these data will yield an optimized and tailored version of MEG-PLAN. It may also be helpful to explore whether motion training components established for MRI technology (e.g., motion potentiometers attached to mock scanners) could be adapted to MEG technology to further enhance participation preparation and success.

Finally, in the service of broadening inclusion in neuroimaging research, every effort will be made to disseminate and make MEG-PLAN protocol resources publicly available. We have started with publication in an open access journal and the provision of protocol documents as additional files, making as much available to the research community as possible. We are also part of a larger network of researchers and clinicians who convened across 3 years (2017–2019) for special interest group meetings at the International Society for Autism Research Annual Meeting (contact corresponding author regarding access to the website and listserv generated from these meetings).

While MEG-PLAN is designed for use with MEG technology and implemented within an academic medical center, the conceptual model (Fig. 1) is generalizable in ways similar to previous protocols developed for MRI. All of the clinical and behavioral concepts can be applied with any technology and in any environment—using parents and providers as partners, maximizing systematic desensitization and habituation to reduce distress, leveraging differential reinforcement to shape cooperation and engagement with scan procedures, using visual support resources to augment communication, and individually tailoring strategies and materials. On the technical side, motion detection and compensation strategies will be unique to each neuroimaging technology, but paradigms can be optimized as a series of short tasks with built-in breaks as well as being passive presentations (with no task demands) when possible. With any technology or environment, stakeholder guidance from parents, providers, self-advocates, and others will assist the development and implementation of any scan support protocols.

## Conclusions

Promising efficacy of MEG-PLAN with the collection of reliable MEG data as shown in the present study suggests a number of future directions. As a second phase of the present study, data collection is already underway for utilizing MEG-PLAN to examine neural activity in youth who have intellectual or developmental disability of varying etiologies (e.g., genetic syndromes). Although initially designed for youth on the autism spectrum, MEG-PLAN can be easily translated into use with other populations of children with limited language and/or cognitive abilities as well as children who may have significant anxiety about undergoing neuroimaging or a medical procedure. Furthermore, adaptation of MEG-PLAN for younger children offers the opportunity for examination of neural markers earlier in development [69] as well as the opportunity for longitudinal studies to evaluate change over time [70]. Whereas the present study utilized age-matched MRI templates for source modeling, the combination of MEG-PLAN and procedures already documented for MRI (e.g., [26, 27]) offers future opportunities for multimodal research with a previously underrepresented group of children who have ASD.

## Supplementary Information

**Additional file 1.** MEG-PLAN Pre-Visit Intake Interview. The Pre-Visit Intake Interview is a semi-structured interview conducted with parents/caregivers during the screening process to support preparation for the MEG visit.

**Additional file 2.** Three Phases of MEG-PLAN Implementation. The table below describes each phase of the MEG-PLAN process, the activities that occur in each phase, and the personnel involved in each phase.

## Data Availability

The datasets analyzed during the current study are available from the corresponding author on reasonable request.

## Footnotes

1 Individuals on the autism spectrum, their parents, and professionals in the field differ regarding the use of person-first (e.g., children with ASD) or identity first (e.g., autistic child) language [1]. With respect for divided opinions, both approaches to terminology are used.
