# Supplementary material for: MEG-PLAN: a clinical and technical protocol for obtaining magnetoencephalography data in minimally verbal or nonverbal children who have autism spectrum disorder
Source: J Neurodev Disord. 2021 Jan 23;13:8. doi: 10.1186/s11689-020-09350-1 (PMC7827989; doi:10.1186/s11689-020-09350-1)
Supplement: Supplementary file 1 — Additional file 1. MEG-PLAN Pre-Visit Intake Interview. The Pre-Visit Intake Interview is a semi-structured interview conducted with parents/caregivers during the screening process to support preparation for the MEG visit. [file 11689_2020_9350_MOESM1_ESM.pdf]

MEG-PLAN PRE-VISIT INTAKE INTERVIEW

CONFIDENTIAL

This intake questionnaire is conducted as a semi-structured interview over the phone with parents/caregivers during the recruitment and screening process. It is conducted by the study coordinator and/or behavior specialist. Questions should be used as a starting point for conversations to understand the child and to prepare accordingly for the MEG visit. Note that this interview follows an initial screening and background history process and thus does not represent the entirety of background information collected on a child participating in a research study.

COMMUNICATION AND ANXIETY

What is the best way to communicate with your child (e.g., when we need to provide instructions or ask him/her to do something)?) – short/simple phrases, visuals, show him/her what to do?
_____

Does your child have trouble acclimating to a new environment?
Yes ☐ No ☐ _____

If yes, is your child typically able to acclimate within the first visit to somewhere new? Or would your child need a second visit? _____

How does your child do with new people? _____

Does your child get anxious? Yes ☐ No ☐ _____
What happens when your child gets anxious/stressed? _____
What helps calm your child down? _____

Does your child have any fears? _____

Does your child dislike being in a hospital environment or have a reaction to doctors wearing white coats? Yes ☐ No ☐ _____

ATTENTION

How long can your child sit and attend to a non-preferred activity without reminders/prompts (e.g., work at table, dentist, haircut)? _____

With reminders/prompts? _____

How long can your child sit and attend to preferred activities (e.g., movies, video games, reading)? _____

What are some things that can help maintain your child's attention (certain toy, movie, etc.)?
_____

REINFORCEMENT AND REWARDS

What are some things your child likes to work for as rewards? _____

Does your child have any special interests (e.g., elevators, TV show, character)?
Yes ☐ No ☐ _____

What are activities the team can do with your child that he/she enjoys? (e.g., bubble play, read a story, etc.) _____

Does your child have any reward systems that are used at school/home? Yes ☐ No ☐
Describe the reward system: _____

TRANSITIONS

Does your child have difficulty…

transitioning from one activity to another?	Yes ☐	No ☐	_____
transitioning from preferred to non-preferred activities?	Yes ☐	No ☐	_____

How long does a transition typically take for your child? _____

What types of strategies do you use to help your child with transitions? (e.g., first-then, visual schedule, timer) _____

CHALLENGING BEHAVIORS

Does your child have a FBA (functional behavior assessment) or behavior plan?

Yes ☐ No ☐ _____

If so, would you be willing to send us a copy? _____

Does your child have self-stimulatory or stimming behaviors (e.g., hand flapping, rocking back and forth)? Frequency? What prompts it?

	Yes ☐	No ☐	_____
Vocal stims?	Yes ☐	No ☐	_____
Echolalia?	Yes ☐	No ☐	_____

Is there anything you can do to interrupt your child's stimming once it starts? _____

How does your child typically react when a demand is placed on him/her (e.g., you tell them to do something)? _____

Does your child display any challenging behaviors? (see table on page 3)

Behavior	Antecedent	Consequence	Duration	Intensity	Frequency
SIB					
Vocal Aggression					
Physical Aggression					
Destruction					
Elopement					
Meltdowns					
Safety Concerns (e.g., mouthing or grabbing objects)					
Other					

Thinking across each of the challenging behaviors we've just discussed, are there any general signs that tell you he/she is going to engage in any of these behaviors? How can you tell that he/she is getting upset? _____

What is your child like after any of these behaviors occur? (e.g., how long does it take to recover, does your child generally feel better to resume activities after a short break?) _____

<u>GENERAL QUESTIONS ABOUT PLANNING FOR THE IMAGING VISIT</u>

The MEG machine has the option of being in a laying down, bed-like position or a sitting up, chair-like position. Do you think NAME would prefer to be laying down or sitting up? _____

There are a lot of experiences involved in the MEG scan that are new for children. I have a list of some of the steps in the process that might be challenging for some children. We will go over the list to identify potential stressors for your child, and then we can talk about some strategies we can use to help your child.

Laying/sitting down on the MEG bed	Yes ☐	No ☐	_____
Scooting his/her head into the helmet	Yes ☐	No ☐	_____
Laying/sitting still	Yes ☐	No ☐	_____
Staying quiet	Yes ☐	No ☐	_____

<u>Sensory issues:</u>

ID bracelet	Yes ☐	No ☐	_____
Scrubs	Yes ☐	No ☐	_____
Alcohol wipes	Yes ☐	No ☐	_____
Glasses	Yes ☐	No ☐	_____
Drawing dots on face	Yes ☐	No ☐	_____
Wand (Digitizing)	Yes ☐	No ☐	_____
Stickers, wires, tape	Yes ☐	No ☐	_____
Gel on stickers	Yes ☐	No ☐	_____
Seatbelt/blanket	Yes ☐	No ☐	_____
Helmet	Yes ☐	No ☐	_____
Listening to beeps	Yes ☐	No ☐	_____

What types of movies does your child like to watch? _____

Your child can watch a movie while he/she is in the MEG scanner. Because your child has to listen to beeps, the movie will be played without sound.

Would your child have difficulty watching his/her favorite movie without sound?
Yes ☐ No ☐ _____

If yes – is it better to watch a new (unfamiliar) movie without sound?
Yes ☐ No ☐ _____

Is your child the type of child who zones out and gets quiet and still when watching a video?
Yes ☐ No ☐ _____

How does your child do with multiple people in the room? _____

Ask parent what would be best to help their child _____

Visual schedule	Yes ☐	No ☐	_____	Overview	Step-by-Step
Timer	Yes ☐	No ☐	_____		
Social story	Yes ☐	No ☐	_____		
Edibles	Yes ☐	No ☐	_____		
Practice visit	Yes ☐	No ☐	_____		
Token board	Yes ☐	No ☐	_____		
First-then board	Yes ☐	No ☐	_____		
High fives	Yes ☐	No ☐	_____		
Verbal praise	Yes ☐	No ☐	_____		
Stickers	Yes ☐	No ☐	_____		
Toys (ask what kind)	Yes ☐	No ☐	_____		
Weighted blanket	Yes ☐	No ☐	_____		

Other: _____
