# Supplementary material for: MEG-PLAN: a clinical and technical protocol for obtaining magnetoencephalography data in minimally verbal or nonverbal children who have autism spectrum disorder
Source: J Neurodev Disord. 2021 Jan 23;13:8. doi: 10.1186/s11689-020-09350-1 (PMC7827989; doi:10.1186/s11689-020-09350-1)
Supplement: Supplementary file 2 — Additional file 2. Three Phases of MEG-PLAN Implementation. The table below describes each phase of the MEG-PLAN process, the activities that occur in each phase, and the personnel involved in each phase. [file 11689_2020_9350_MOESM2_ESM.pdf]

Three Phases of MEG-PLAN Implementation

**1. Assessment**		
Phase	***Phase Description***	***Phase Personnel***
Behavioral Assessment	Prior to the phenotyping visit, the clinical team works with parents and caregivers to collect information about the participant. Parents are first asked to watch a video (49) to become familiar with the MEG process prior to completing an intake interview with the behavior specialist. Information about challenging behaviors and triggers, communication strategies, and preferred reinforcers (e.g., favorite movies, special interests) is collected. This information helps the clinical team identify signs of distress, provide proactive breaks to prevent challenging behaviors, build rapport with the participant using preferred motivators to reinforce positive behaviors, and identify motivators for differential reinforcement during the scan.	Behavior Specialist<br>Parent/Provider
Phenotyping Visit Summary	During the phenotyping visit, participants are assessed to characterize nonverbal cognitive and language ability as well as confirm ASD diagnosis and minimally verbal status. Participants are assessed by clinicians with significant experience working with minimally verbal/nonverbal individuals with ASD and applied behavior analytic strategies. The phenotyping visit helps further identify challenges for the MEG visit, and provides an opportunity for the clinician/behavior specialist to start to build rapport with the child. During the MEG visit, the clinician or behavior Specialist who work with the participant at the phenotyping visit are present to provide behavioral support.	Clinician<br>Behavior Specialist
**2. Plan and Preparation**		
Phase	***Phase Description***	***Phase Personnel***
MEG Clinical Support	Based on parent input and information collected during the phenotyping visit, an individualized plan tailored to each participant is developed. The clinical support plan comprises a practice "kit" provided to parents, a summary of preferred reinforcers and helpful strategies (based on both parent report and observation during the phenotyping visit), a list of triggers/antecedents to challenging behaviors, preferred videos that can be used during the MEG scan, whether the participant will be lying or sitting for the MEG scan, and other notes.	Behavior Specialist
Home Practice and Preparation	Parents are given a practice kit to help them expose the participant to specific sensory challenges (e.g., practice "wires" for coils) prior to the MEG visit. The practice kit included handouts providing information about MEG, general information for practicing with children, and individualized practice plans based on the challenges anticipated during the intake interview. Each practice plan describes what the process looks like, why each step is important, pictures showing each step, and materials needed for practicing. Materials not easily accessible to parents (e.g., alcohol wipes, plastic string to simulate coils) are provided with the handout (see Figure 4). Parents are encouraged to practice in the weeks leading up to the MEG visit.	Parent/Provider

3. MEG Visit		
Phase	***Phase Description***	***Phase Personnel***
MEG Clinical Components	MEG-PLAN is implemented as planned in the second phase. Although the MEG protocol takes up to an hour to collect, the visit is scheduled for three hours to provide sufficient time to allow the participant to habituate to the MEG environment and the materials. The behavior specialist and parent/provider are in the room with the participant during data collection to provide reminders to stay still and help the participant remain comfortable during data collection.	Behavior Specialist<br>MEG Technologist<br>Imaging Research Assistant<br>Parent/Provider
MEG Technical Components	As designed, the MEG paradigms presented during the visit are both passive and optimized to minimize length and incorporate opportunities for breaks. Continuous motion detection via active coils is implemented, allowing for modestly increased head motion.	MEG Technologist<br>Imaging Research Assistant
